# Complications and Related Risk Factors of Peripherally Inserted Central Catheters in Neonates: A Historical Cohort Study

**DOI:** 10.34172/aim.2023.33

**Published:** 2023-04-01

**Authors:** Seyyed Mostajab Razavinejad, Najib Saeed, Shahnaz Pourarian, Mehrdad Rezaei, Reza Bahrami, Negar Yazdani, Hamide Barzegar, Fatemeh Yarmahmoodi

**Affiliations:** ^1^Department of Pediatrics, Division of Neonatology, Neonatal Research Center, Shiraz University of Medical Sciences, Shiraz, Iran; ^2^Department of Pediatric, School of Medicine, Shiraz University of Medical Sciences, Shiraz, Iran; ^3^Department of Nursing, Neonatal Research Center, Shiraz University of Medical Sciences, Shiraz, Iran; ^4^Department of Radiology, Medical Imaging Research Center, Shiraz University of Medical Sciences, Shiraz, Iran

**Keywords:** Bacteremia, Catheterization, Infant, Premature birth, Risk factors

## Abstract

**Background:**

Peripherally inserted central catheters (PICCs) are an effective method for medication and nutrition infusion in preterm neonates. The present study aimed to identify the incidence of the most common complications of PICC implantation and evaluate the risk factors of each complication.

**Methods:**

This historical cohort study was conducted on 2500 neonates with birth weights (BWs)≥500 g and gestational age (GA)>24 weeks who had a history of PICC inserted in three NICUs between August 2015 and August 2018. Data were collected by reviewing medical records. Demographic data and indices of catheter placement, duration of catheter placement, and common complications were recorded. Data analysis was done using SPSS-21.

**Results:**

The median cubital vein had the most PICC placement (43%). The most common complication was tip malposition (48.2%). The incidence rates (95% CI) of the main complications such as malposition, edema/occlusion, and PICC migration were 0.0356 (0.0337-0.0377), 0.0134 (0.0122-0.0147), and 0.0088 (0.0079-0.0099), respectively. PICC insertion position was the strongest predictor of malposition for the cephalic vein. Besides, the incidence of malformation in the cephalic vein was about six times higher than in the median cubital vein. Independent risk factors for non-technical complications included BW (OR=0.59, 95% CI 0.44-0.79), administration of hyperosmolar medications (OR=3.43, 95% CI 2.62-4.51), position (OR=2.43, 95% CI 1.92- 3.08), and duration of catheter presence (OR=1.02, 95% CI 1.01-1.03) (*P*<0.001).

**Conclusion:**

The most common complication was malposition related to catheter placement in an emergency. Moreover, BW, administration of hyperosmolar medications, and duration of catheter presence were the most critical risk factors for non-technical complications. Therefore, it is recommended to educate the PICC insertion team to reduce tip malposition and replace long-term catheters.

## Introduction

 Fifteen million premature infants are born annually, and this rate is rising worldwide. Complications of preterm birth were the principal reason for one million deaths amongst children under five in 2015.^1,2^ Furthermore, preterm birth is associated with disabilities, including learning disabilities and visual and hearing problems.^3^

 The health care providers, specifically newborn intensive care units (NICUs), are routinely forced to provide care, often parenteral nutrition and intravenous medications, for preterm neonates until they become independent. This advanced care is typically performed by placing central catheters, which serve as a lifeline in an intensive care unit, and are often used in the NICU. The centrally located catheters utilized in NICUs include umbilical venous catheters, umbilical arterial catheters,^4^ and peripherally inserted central catheter (PICC) lines.^5^ PICCs reduce pain caused by the frequent intervention of peripheral intravenous cannulation and allow for safe administration of nutrients or concentrated intravenous fluids.^6^

 Even though PICC insertion prevents complications, including neck and chest vessel injuries and pneumothorax, PICCs are linked to several complications such as local irritability and fatal catheter-related bloodstream infections.^7^ Furthermore, catheter malplacement can result in sudden removal, tip migration, and fracture. These complications may lead to the need for premature removal of the current PICC and reinsertion of another line that can expose the individual to further risks and complications.

 Numerous researchers have considered the incidence of these complications.^8^ However, most were questionnaire-based or single-center, which were done more often in adults compared to children or infants.

 Despite many medical advances, premature birth is still a universal problem in developing and developed countries.^2^ Recent literature in Iran showed an increasing prevalence of prematurity, from 9.2% to 10% in 2015 and 2017, respectively,^9,10^ while there are limited studies on the prevalence of PICC complications in neonatal populations and no evidence of possible risk factors for PICC complications. Therefore, the present study aimed to identify the incidence of the most common complications of PICC insertion at NICUs and to evaluate the risk factors for these complications.

 This study was conducted according to the STROBE reporting checklist.

## Materials and Methods

###  Design and Subjects of the Study 

 This historical cohort study was conducted on 2500 neonates who had a history of PICC inserted in NICUs. Three NICUs of Shiraz University of Medical Sciences hospitals, which had access to medical records of eligible neonates, provided data.

 In this study, we included all neonates with gestational age (GA) over 24 weeks and birth weight (BW) ≥ 500 g who had a PICC inserted between August 2015 to August 2018. Neonates with fatal congenital abnormalities or significant chromosomal defects were excluded. Demographic information, indices of catheter placement, duration of catheter placement, and common complications were collected by reviewing the patients’ records. In our study, extremely low BW was defined as BW < 1000 g, and very-low BW was defined as BW < 1500 g.

###  Technical Information 

 PICC insertion was performed by standard sterile methods by neonatal fellows or the nurse PICC team without administration of prophylactic antibiotics. The polyurethane central venous catheter (Nutriline and PremiCath, Vygon Corp., Aachen, Germany) and needle systems that were used, consisted of 1 Fr catheters with 27-gauge needles.

 This procedure was done on the bed. Post-insertion chest radiographs were taken with the limbs in standard resting position to evaluate the proper position (anteroposterior and lateral views in which the arm is positioned at about 30-45 degrees to the body). According to the following criteria, PICC line tips were defined as in the proper position. Tip location should be in the superior vena cava (SVC) to the right atrium junction that is 0.5-1 cm and 1-2 cm outside of the cardiac chambers in premature and older infants, respectively.^11^ Our clinical goal was placement of the catheter tip in the SVC but outside right atrium. Catheters outside the SVC were discontinued or repositioned based on the neonatologist’s opinion. In all PICCs, as per standard unit policy, heparin was injected at 0.5 units/kg/h. Patients’ charts documented the details of catheter insertion, removal, and hourly status of catheter site and infusion volume. The neonates’ nurse checked the PICC dressing daily and changed it if it was dirty or loose or if bleeding occurred.

 All catheters were discontinued after the completion of fluid therapy and the start of enteral feeding (150–160 mL/kg/d). Premature removal was inevitable if complications occurred.

 Peripheral blood culture was obtained from suspected sepsis cases. An attending neonatologist also took central blood culture. In premature neonates, blood sampling from small central catheters was often not possible after days of intravenous therapy. In cases with positive blood culture and a central line, a peripheral blood culture was always done 48 h after antibiotic therapy.

 The crude incidence rate for each complication was calculated as the ratio of subjects experiencing that specific complication to the total number of patients. Two additional variables were created describing the total number of complications per subject and whether a patient experienced at least one complication during the dwelling time. Incidence rates per 1000 catheter-days were also calculated based on the following formula:


$$Incidence\;Rate\;per\;Catheter\;Days=\frac{Frequency\;of\;Patients\;with\;a\;Complication}{Total\;Catheter\;Days}$$

Total catheter-days are represented as follows:


$$\sum_{1}^{i=n}\left(fi\times di\right)$$

which n = duration of indwelling catheters in day-unit; d = dwell time patients tolerated a catheter; and

 f = frequency of patients with d units of the catheter.

 In our study, the primary outcomes were identifying the incidence and type of PICC complications. Based on the literature review, we identified nine complications and gathered information regarding each complication for all included subjects:

- Catheter migration is the displacement of the catheter tip more than 2 cm from its primary position, determined by a pre-post chest radiograph. The catheter insertion length is measured from its entry point to the right sternoclavicular joint and perpendicular to the sternum.
- Catheter-related bloodstream infection (CRBSI) is the observation of at least one peripheral positive blood culture associated with some clinical symptoms, including fever ( > 38 ^o^C), hypothermia ( < 36 ^o^C), apnea, or bradycardia when PICC is appropriately located.
- Malposition is the placement of tip catheters outside the central vena cava.
- Edema/occlusion is a tissue bulge caused by the accumulation of fluid around the catheter insertion point that can prevent fluid infusion, leading to the removal of the catheter.
- Phlebitis is the venous endothelium irritation resulting from the catheter, which is associated with symptoms such as swelling, pain, erythema, tenderness, warmth of the area, and palpable venous cord confirmed by sonography.
- Pleural Effusion: Ultrasound evaluations and standard anterior-posterior chest x-rays were performed in case clinical symptoms were suggestive. Results were evaluated by either radiologists, attending physicians or neonatal intensivists where appropriate.
- Bleeding from insertion site: Oozing from the insertion site within 24 hours of PICC insertion requiring more than five minutes of pressure on the puncture site.
- On ultrasonography catheter-related thrombosis associated with clinical symptoms, including swelling, pain, erythema, and vein obstruction by a blood clot.
- Arrhythmia: During the insertion procedure, the detection of atrial or ventricular dysrhythmias in bradycardia, tachycardia, or supraventricular tachycardia.

Moreover, dwell time was considered the time interval between the dates of insertion and removal of the catheter.

###  Statistical Analysis 

 The Statistical Package for Social Sciences (SPSS, version 18, Chicago, Illinois) was used for data analysis, and Stata MP version 15 for graphical representation of the data and models. Data normality was assessed by Kolmogorov–Smirnov test. Descriptive data were summarized using incidence rate (95% confidence intervals [CI]), mean and standard deviation (SD), median and interquartile range (IQR), and frequency (%). In addition, analytical data were analyzed by univariate logistic regression, developing multivariable logistic regression models, univariate analyses, and the likelihood ratio or Hosmer-Lemeshow test. A *P* value less than 0.05 was considered statistically significant for all tests.

## Results

###  Clinical Characteristics

 Two thousand and five hundred neonates were included with a mean GA of 32 ± 3 weeks and a median (IQR) BW of 1600 [1200-2190] grams. Most cases were premature neonates admitted for receiving total parenteral nutrition (TPN) (51%). All the PICCs were inserted in the upper extremity veins. Among the upper extremity veins, such as the basilic, median cubital, and cephalic vessels, the median cubital vein had the greatest number of PICC placements (43%). Intravenous antibiotics were used in 32.8% of neonates, while only 1.6% received anticonvulsant medications. Catheter dwell time was reported in days with a positively skewed non-normal distribution. The mortality rate was 205 (8.2%), of which 45 (1.8%) were PICC-related. There were 2500 neonates, giving rise to 33750 catheter days (30-2900). Table 1 summarizes the baseline demographics of the participants.

**Table 1:** Baseline Demographics of the Studied Population

**Variables**	**No. (%)**	**Mean±SD**	**Median [IQR]**	**Range (Min-Max)**
Gestational age (wk)	—	32 ± 3	32 [30-34]	17 (24-41)
Gestational age categories (wk)				
< 28	395 (15.8%)	—	—	—
28-34	1540 (61.6%)	—	—	—
≥ 35	565 (22.6%)	—	—	—
Birth weight (g)	—	1745 ± 719	1600 [1200-2190]	4250 (550-4800)
Categories of birth weight (g)				
< 1000	400 (16.0%)	—	—	—
1000-1500	705 (28.2%)	—	—	—
1500-2000	645 (25.8%)	—	—	—
2000-2500	435 (17.4%)	—	—	—
≥ 2500	315 (12.6%)	—	—	—
Total parenteral nutrition	1275 (51%)	—	—	—
Hyperosmolar medications	835 (33.4%)	—	—	—
IV Antibiotics^a^	820 (32.8%)	—	—	—
IV Anticonvulsant^b^	40 (1.6%)	—	—	—
PICC insertion site				
Basilic vein	490 (19.6%)	—	—	—
Median cubital vein	1075 (43.0%)	—	—	—
Cephalic vein	935 (37.4%)	—	—	—
Duration of catheter (days)	—	14 ± 13	9 [5-18]	69 (1-70)
Mortality rate	205 (8.2%)	—	—	—
Related to PICC	45 (1.8%)	—	—	—
Not related to PICC	160 (6.4%)	—	—	—

PICC, Peripherally inserted central catheter; SD, Standard deviation; IQR, Interquartile range. ^a^Vancomycin Amphotericin; ^b^Phenytoin.

###  PICC-Related Complications and Incidence Rates

 Malposition was the most common adverse outcome of PICC insertion (48%). This outcome was followed by edema/occlusion and PICC migration. CRBSIs were reported at 6.2%. Arrhythmia, pleural effusion, and catheter-related thrombosis were infrequent complications, accounting for less than 1% of the patients.

 Overall, 250 (9.2%) patients had positive blood cultures, and coagulase-negative *Staphylococcus aureus* was the most common isolated pathogen (84%) (Table2). Table 3 shows all the complication incidence rates with their related 95% CI s.

**Table 2:** PICC-Related Complications, Crude Incidence, and Incidence/1000 Catheter-Days

**Type of Complication**	**Crude Incidence**	**Incidence/1000 Catheter-Days**
Malposition	1205 (48.2%)	35.70
Edema/Occlusion	455 (18.2%)	13.48
PICC migration (dislodged)	300 (12%)	8.89
Positive blood cultures	230 (9.2%)	6.81
Coagulase-Negative *Staphylococcus aureus *	210 (8.4%)	6.22
*Candida albicans*	35 (1.4%)	1.03
*Staphylococcus epidermidis*	5 (1.2%)	0.15
Positive tip of PICC culture	190 (7.6%)	5.63
Cather-related blood stream infection	155 (6.2%)	4.59
Phlebitis	90 (3.6%)	2.66
Bleeding from PICC insertion site	20 (0.8%)	0.60
Arrhythmia	10 (0.4%)	0.30
Pleural effusion	10 (0.4%)	0.30
Catheter-related thrombosis	5 (0.2%)	0.15
At least one complication	1455 (58.2%)	43.11

PICC, peripherally inserted central catheter.

**Table 3:** PICC-related Complications, Incidence rate with 95% Confidence Interval

**Type of Complication**	**Incidence Rate**	**95% CI**
Malposition	0.0356	(0.0337-0.0377)
Edema/Occlusion	0.0134	(0.0122-0.0147)
PICC migration (dislodged)	0.0088	(0.0079-0.0099)
Positive blood cultures	0.0074	(0.0065-0.0083)
Positive tip of PICC culture	0.0056	(0.0048-0.0064)
Cather-related blood stream infection	0.0068	(0.0059-0.0077)
Phlebitis	0.0026	(0.0021-0.0032)
Bleeding from PICC insertion site	0.0005	(0.0003-0.0009)
Arrhythmia	0.0002	(0.000-0.0005)
Pleural effusion	0.0002	(0.0001-0.0005)
Catheter-related thrombosis	0.0001	(0.0000-0.0003)
At least one complication	0.0715	(0.0687-0.0744)

PICC, peripherally inserted central catheter; CI, confidence interval.

###  Malposition

**Figure 1:**
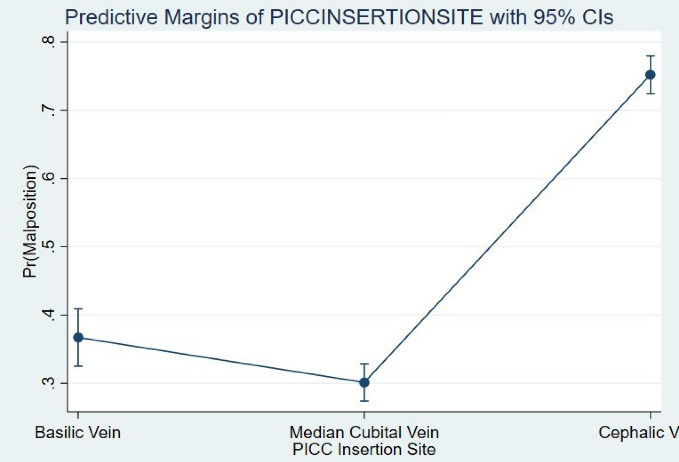
Comparison of Adjusted Probabilities of Malposition Between Various Insertion Sites.

 There was a strong relationship between the PICC insertion site and malposition. After controlling the covariates, the cephalic vein (OR [95% CI]: 7.56 [6.16-9.28], *P* < 0.001) was associated with 6-fold higher odds of malposition compared with the basilic vein (OR [95% CI]: 1.36 [1.07-1.71, *P* = 0.009) (Figure 1). In the presence of the PICC insertion site, none of the baseline independent variables served as significant contributors to malposition.

###  Catheter-Related Blood Stream Infections 

 In our study, BW, TPN, and dwelling time were significant predictors of CRBSI. BW and GA were inversely related to the probability of CRBSI. Neonates who received TPN or used anticonvulsants showed significantly higher odds of CRBSI. PICCs placed in the cephalic vein also showed a higher risk of being complicated by infections. Moreover, infants with higher indwelling time were at increased risk of infection. PICC dwell time was the strongest independent predictor of CRBSI, such that each one-day increase in PICC duration was associated with an 11% increase in the odds of developing CRBSI. This relationship was independent of GA, BW, and administration of TPN or anticonvulsants. Phenytoin and parenteral nutrition were also significant predictors of CRBSI, i.e. patients who received phenytoin or TPN were respectively 10 and 8.6 times more likely to suffer from CRBSI. Table 4 presents the associated regression coefficients, odds ratios, and 95% CI s from the unadjusted and controlled analyses.

**Table 4:** Catheter-Related Blood Stream Infection and Associated Risk Factors

**Predictors**	**Univariate Analysis**				**Multivariate Analysis**			
	**OR (95% CI) **	**SE **	**Z**	***P*** ** Value**	**OR (95% CI) **	**SE **	**Z**	***P*** ** Value**
Gestational age (wk)	0.72 (0.68-0.77)	0.031	-10.44	< 0.001	0.96 (0.87-1.07)	0.052	-0.61	0.54
Birth weight (kg)	0.17 (0.12-0.26)	0.19	-8.85	< 0.001	1.86 (0.95-3.64)	0.342	1.82	0.07
TPN	35.44 (14.48-86.71)	0.456	7.82	< 0.001	8.60 (2.83-26.1)	0.566	3.8	< 0.001
Anticonvulsant	5.32 (2.55-11.10)	0.375	4.46	< 0.001	10.5 (4.34-25.6)	0.453	5.2	< 0.001
Dwell time (days)	1.12 (1.11-1.13)	0.006	17.82	< 0.001	1.11 (1.09-1.12)	0.007	13.86	< 0.001
PICC insertion site			2.18	0.09				
Median cubital		Reference				Reference		
Basilic	1.20 (0.76-1.90)	0.233	0.79	0.34	1.18 (0.66-2.13)	0.298	0.58	0.56
Cephalic vein	1.49 (1.03-2.15)	0.186	2.16	0.03	1.36 (0.78-2.35)	0.28	1.1	0.27

PICC, peripherally inserted central catheter; TPN, total parenteral nutrition; OR, Odd ratio; SE, Standard error; Z, Z score.

###  Complications Excluding Malposition (At Least One Complication)

 Most patients (48%) experienced malposition; they experienced at least one complication, excluding malposition. As shown in Table 5, all independent variables were significantly related to developing non-technical complications in univariate analysis.

**Table 5:** Non-technical Complications of PICC Insertion and Associated Risk Factors

**Variables**	**Univariate Analysis**				**Multivariate Analysis**			
	**OR (95% CI)**	**SE**	**Z **	***P*** ** Value**	**OR (95% CI)**	**SE**	**Z **	***P*** ** Value**
Gestational age (wk)	0.77 (0.74-0.79)	0.01	15.95	< 0.001	0.98 (0.93-1.03)	0.03	-0.56	0.579
Weight (kg)	0.22 (0.18-0.27)	0.02	16.06	< 0.001	0.59 (0.44-0.79)	0.09	-3.48	< 0.001
**Insertion site**								
Median cubital	Reference				Reference			
Basilic vein	1.81 (1.40-2.33)	0.23	4.60	< 0.001	1.20 (0.90-1.61)	0.18	1.26	0.206
Cephalic vein	3.50 (2.85-4.29)	0.37	11.99	< 0.001	2.21 (1.72-2.84)	0.28	6.24	< 0.001
Malposition	2.62 (2.19-3.14)	0.24	10.50	< 0.001	2.43 (1.92-3.08)	0.29	7.43	< 0.001
TPN	6.78 (5.51-8.33)	0.71	18.20	< 0.001	1.32 (0.93-1.88)	0.24	1.59	0.112
Hyperosmolar medication	7.46 (6.15-9.04)	0.73	20.46	< 0.001	3.43 (2.62-4.51)	0.48	8.93	< 0.001
Dwell time (Days)	1.05 (1.04-1.05)	0.00	14.00	< 0.001	1.02 (1.01-1.03)	0.00	5.89	< 0.001

PICC, peripherally inserted central catheter; TPN, total parenteral nutrition; OR, Odd ratio; SE, Standard error; Z, Z score.

 Our results showed that malposition and hyperosmolar medications were the most significant risk factors for developing non-technical complications. Our results indicated a non-significant effect for GA and TPN on non-technical complications. PICC insertion site remained a strong predictor of complications; for example, the probability of developing at least one complication in neonates with a PICC in the cephalic vein was 2.2 times that of those with PICCs in the median cubital vein (OR: 2.2, 95% CI: 1.72-2.84). The total incidence of complications of PICC increased significantly.

## Discussion

 This historical cohort study on the medical records of 2500 neonates showed that malposition, occlusion, and CRBSI are the most common complications associated with PICC insertion. PICC insertion occurred in an emergency due to many admissions when no other venous access was available. PICC placement was essentially the responsibility of trained nurses in our NICUs. Some studies suggest that the nurses’ knowledge and attitude can directly influence the rate of complications, especially malposition.^11^ We propose that the neonatal PICC insertion team should incorporate physicians and nurses to improve placement standards and post-insertion care. In this study, the PICC insertion site was the strongest predictor of malposition irrespective of neonates’ weight, GA, and whether TPN or hyperosmolar infusions were indicated. In contrast to the median cubital vein, the cephalic vein was associated with approximately 7.5 times higher chance of malposition. This observation may be explained by the anatomical variations of the neonates regarding the cephalic vein in the upper arm, which ends in small vessels, making it difficult or even impossible to direct the catheter to a central location.^12^ It should also be mentioned that inserting a catheter via a cephalic vein with its higher risk of malposition leads to other complications, including mechanical infection and infiltration.^13^ Thus, it is suggested that PICCs should be placed via the median cubital vein and their location should be assessed post insertion through bedside ultrasound or X-ray techniques to prevent most complications.^14^ In addition, occlusion was another complication in our centers. In various reports, it ranged from 2-36%^15^ and was reported as the most common cause of catheter removal.^16,17^

 In our study, delivering hyperosmolar medication through PICC, choosing the cephalic vein as the insertion site, and having longer dwell times were the most substantial contributors to the development of at least one complication during the NICU stay. Hyperosmolar solutions are associated with complications such as phlebitis, extravasation, leaking, and occlusion.^18^

 Another common complication of PICC insertion was CRBSI at the rate of 4.59 episodes per 1000 catheter days. The incidence of CRBSI infection ranges from 0 to 15 per 1000 catheter days in various reports.^18-21^ In Iran, a study reported 5.8 cases of central catheter-related bloodstream infections in 1000 catheter days in pediatrics in six hospitals.^21^ Other studies that estimated CRBSI in 15 000 patients in 50 countries and Iran reported a value of four cases per 1000 line days, which is consistent with our findings.^22,23^ Chang et al showed that CRBSI is one of the rare complications of PICC.^24^ The low CRBSI incidence can be related to lower sensitivity for detecting infections or higher quality of care in our centers. A report from one NICU at Covenant Healthcare in Saginaw, Michigan, showed zero incidence of catheter-related infections; since it signified the importance of implementing nosocomial infection preventive strategies,^23^ the use of a standard guideline was suggested in our NICUs.

 Infusion of hyperosmolar medications and TPN were the most decisive independent risk factors for developing CRBSI. The lipid component of TPN is the constituent predisposing factor for CRBSI.^25^ Although TPN administration has been a fundamental risk factor of CRBSI,^15^ the effect size varies from 2-16 in various subsets of studied neonates. Therefore, it is proposed that clinicians minimize TPN administration and save the remedy for neonates with definite indications.

 Consistent with our findings, BW is an independent risk factor for CRBSI; this might be explained by the fact that neonates with VLBW are susceptible to infections because they have an immature immune system, have repeated contact with hospital personnel, and undergo invasive procedures.^5^

 Since a lengthy hospitalization can increase the risk of infection, neonates with lower GA should inherently carry a higher risk of CRBSI. Even after adjusting for BW and GA, our findings indicated an 11% increase rate of CRBSI per one-day increase in the length of hospitalization. This study was consistent with two studies from the United States, revealing an increased independent risk of CRBSI after the 36th day of hospitalization.^18^ Contradictory studies mentioned that the risk of infection was significant for a dwell time of up to 7 days.^26,27^ We propose that for neonates hospitalized for longer than four weeks with a PICC, at least one blood culture per week should be performed.

 In our centers, coagulase-negative *Staphylococcus aureus* followed by *Candida* species accounted for 84% and 14% of laboratory-confirmed bloodstream infections, respectively. This finding is similar to Lee’s study,^5^ a rise in premature neonatal care recently increased the incidence of candidemia. Up to 30% of the isolated pathogens in CRBSI at NICUs have been attributed to *Candida* species.^25^ Our study showed that candidemia occurred in GA < 30 weeks. Therefore, it may be recommended that prophylactic use of antifungal agents (fluconazole) may guarantee a lower incidence of infections if the neonate is extremely premature.

 Eventually, as the neonate reaches the 14th day of hospitalization, he/she gets a 30% chance of at least one complication. Therefore, it is recommended that all the health care team dealing with the premature neonate should be aware of PICC complications. In addition, regular assessment for early detection of these preventable adverse outcomes should be incorporated into the daily care of those neonates expected to have hospital stays beyond two weeks.

 Although it has been claimed in several studies, including in our country, that there is a nationwide infection surveillance program already implemented into the health care system, reports are difficult to find. We propose that strict data gathering and timely trend analysis should become prerequisites for setting up neonatal intensive care units. Moreover, a robust continuous education system regarding complications and precautions for physicians and nurses should be developed and incorporated into the existing framework of premature care.

 Our study has some limitations. First, this historical study hindered us from gathering information regarding possible contributors to PICC complications, including the time difference between PICC insertion and developing one or more complications. Second, since data were collected from the neonates’ medical records, which recorded neonates’ data only once, the possibility of recurrence of complications and the time of their follow up are not available. Third, we did not gather information on the material composition of catheters used in this study. Despite these limitations, our study is the first report on the incidence and type of PICC complications in a large sample of neonates (GA: 24-41 weeks), which guarantees statistical soundness and power required for assessing rare complications. To the best of our knowledge, no studies have addressed this issue, specifically in the neonates in Iran and other countries in the Eastern Mediterranean region.

 In conclusion, the most common complication was malposition related to catheter placement in emergency conditions. Moreover, BW, administration of hyperosmolar medications, and the duration of catheter presence were the most critical risk factors for non-technical complications. Therefore, it is recommended to educate the PICC insertion team, such as physicians and nurses, to reduce tip malposition and replace long-term catheters.

## References

[1] Liu L, Oza S, Hogan D, Chu Y, Perin J, Zhu J (2016). Global, regional, and national causes of under-5 mortality in 2000-15: an updated systematic analysis with implications for the sustainable development goals. Lancet.

[2] Blencowe H, Cousens S, Oestergaard MZ, Chou D, Moller AB, Narwal R (2012). National, regional, and worldwide estimates of preterm birth rates in the year 2010 with time trends since 1990 for selected countries: a systematic analysis and implications. Lancet.

[3] World Health Organization (WHO). March of dimes; the partnership for maternal, newborn & child health; save the children. In: Born too Soon: The Global Action Report on Preterm Birth. WHO; 2012.

[4] Yazdani N, Badfar G, Pourarian S (2020). Evaluation of umbilical vein catheter position in neonates by thoraco-abdominal radiography versus echocardiography. Iran J Pediatr.

[5] Lee JH (2011). Catheter-related bloodstream infections in neonatal intensive care units. Korean J Pediatr.

[6] Legemaat M, Carr PJ, van Rens RM, van Dijk M, Poslawsky IE, van den Hoogen A (2016). Peripheral intravenous cannulation: complication rates in the neonatal population: a multicenter observational study. J Vasc Access.

[7] Chopra V, Flanders SA, Saint S (2012). The problem with peripherally inserted central catheters. JAMA.

[8] Njere I, Islam S, Parish D, Kuna J, Keshtgar AS (2011). Outcome of peripherally inserted central venous catheters in surgical and medical neonates. J Pediatr Surg.

[9] Sharifi N, Khazaeian S, Pakzad R, Fathnezhad Kazemi A, Chehreh H (2017). Investigating the prevalence of preterm birth in Iranian population: a systematic review and meta-analysis. J Caring Sci.

[10] Vakilian K, Ranjbaran M, Khorsandi M, Sharafkhani N, Khodadost M (2015). Prevalence of preterm labor in Iran: a systematic review and meta-analysis. Int J Reprod Biomed.

[11] Sneath N (2010). Are supine chest and abdominal radiographs the best way to confirm PICC placement in neonates?. Neonatal Netw.

[12] Westergaard B, Classen V, Walther-Larsen S (2013). Peripherally inserted central catheters in infants and children - indications, techniques, complications and clinical recommendations. Acta Anaesthesiol Scand.

[13] Jain A, Deshpande P, Shah P (2013). Peripherally inserted central catheter tip position and risk of associated complications in neonates. J Perinatol.

[14] Wrightson DD (2013). Peripherally inserted central catheter complications in neonates with upper versus lower extremity insertion sites. Adv Neonatal Care.

[15] Pettit J (2002). Assessment of infants with peripherally inserted central catheters: part 1Detecting the most frequently occurring complications. Adv Neonatal Care.

[16] Can E, Salihoğlu O, Oztürk A, Güngör A, Güler E, Hatipoğlu S (2014). Complication profiles of central and non-central 1 Fr PICCs in neonates weighing < 1500 g. J Matern Fetal Neonatal Med.

[17] Dongara AR, Patel DV, Nimbalkar SM, Potana N, Nimbalkar AS (2017). Umbilical venous catheter versus peripherally inserted central catheter in neonates: a randomized controlled trial. J Trop Pediatr.

[18] Milstone AM, Reich NG, Advani S, Yuan G, Bryant K, Coffin SE (2013). Catheter dwell time and CLABSIs in neonates with PICCs: a multicenter cohort study. Pediatrics.

[19] Costa P, Kimura AF, Brandon DH, Damiani LP (2016). Predictors of nonelective removal of peripherally inserted central catheters in infants. Biol Res Nurs.

[20] Jahani-Sherafat S, Razaghi M, Rosenthal VD, Tajeddin E, Seyedjavadi S, Rashidan M (2015). Device-associated infection rates and bacterial resistance in six academic teaching hospitals of Iran: findings from the International Nocosomial Infection Control Consortium (INICC). J Infect Public Health.

[21] Eshrati B, Masoumi Asl H, Afhami S, Pezeshki Z, Seifi A (2018). Health care-associated infections in Iran: a national update for the year 2015. Am J Infect Control.

[22] Rosenthal VD, Al-Abdely HM, El-Kholy AA, AlKhawaja SAA, Leblebicioglu H, Mehta Y (2016). International Nosocomial Infection Control Consortium report, data summary of 50 countries for 2010-2015: device-associated module. Am J Infect Control.

[23] Rosenthal VD, Maki DG, Mehta Y, Leblebicioglu H, Memish ZA, Al-Mousa HH (2014). International Nosocomial Infection Control Consortium (INICC) report, data summary of 43 countries for 2007-2012Device-associated module. Am J Infect Control.

[24] Chang LX, Chen YW, Wang MC, Zhao SY, Wang M, Tian Y (2021). Analysis of peripherally inserted central catheter-related complications: a retrospective cohort study of 2,974 children with blood diseases in a single center of China. Ann Palliat Med.

[25] Holmes A, Doré CJ, Saraswatula A, Bamford KB, Richards MS, Coello R (2008). Risk factors and recommendations for rate stratification for surveillance of neonatal healthcare-associated bloodstream infection. J Hosp Infect.

[26] Zingg W, Posfay-Barbe KM, Pfister RE, Touveneau S, Pittet D (2011). Individualized catheter surveillance among neonates: a prospective, 8-year, single-center experience. Infect Control Hosp Epidemiol.

[27] Smith PB, Benjamin DK Jr, Cotten CM, Schultz E, Guo R, Nowell L (2008). Is an increased dwell time of a peripherally inserted catheter associated with an increased risk of bloodstream infection in infants?. Infect Control Hosp Epidemiol.

